# Fire Detection Algorithm for Complex Warehouse Scenarios Based on YOLOv11n

**DOI:** 10.3390/s26206491

**Published:** 2026-10-13

**Authors:** Liwei Guan, Bicai Li, Junjie Lu, Yunping Wu, Wenzao Shi, Zhigao Huang, Yue Chen, Qiuliang Wang

**Affiliations:** 1 College of Physics and Energy, Fujian Normal University, Fuzhou 350117, China; 2 College of Photonic and Electronic Engineering, Fujian Normal University, Fuzhou 350117, China; lbicai2025@163.com (B.L.); lujj352@163.com (J.L.);; 3 Shuili Fire Protection Technology Co., Ltd., Quanzhou 362300, China

**Keywords:** fire detection, YOLOv11, dynamic upsampling, residual multi-scale attention, adaptive downsampling, small object detection

## Abstract

**Highlights:**

**What are the main findings?**

- We proposed FC-YOLO, a lightweight fire detection algorithm based on YOLOv11n, achieving a precision of 91.8% and mAP@0.5 of 90.1% with only 2.2M parameters.
- Successfully integrated a dynamic upsampling module (DySample) and an additional small-object detection layer (P2) to significantly improve the recognition of small-scale flames.

**What are the implications of the main findings?**

- The algorithm effectively reduces computational complexity while maintaining high robustness against complex warehouse background interference.
- Its lightweight design demonstrates strong potential for deployment on edge devices in early warehouse fire warning systems.

**Abstract:**

Fire detection in warehouse scenarios is challenged by complex background interference, weak features of small flame targets, and indistinct flame textures, which can reduce detection performance and constrain model deployment. To address these issues, we propose FC-YOLO (Fire Complexity-YOLO), a lightweight fire detection algorithm based on YOLOv11n. A dynamic upsampling module (DySample) is introduced into the neck network to adaptively adjust feature sampling locations and reduce information loss. An adaptive downsampling module (ADown) is employed in the backbone and neck networks to reduce computational complexity while enabling efficient multi-scale feature extraction. A residual multi-scale attention module (IEMA) is constructed in the neck network to enhance feature representation and focus on critical regions. Finally, an additional small-object detection layer (P2) is introduced to improve small-flame detection. On a self-built flame dataset, FC-YOLO achieves 91.8% precision, 84.7% recall, 90.1% mAP@0.5, and 50.0% mAP@0.5:0.95 with 2.2M parameters. Compared with YOLOv11n, these metrics improve by 1.8%, 4.6%, 2.4%, and 3.7%, respectively, while parameters decrease by 14%. These results demonstrate that FC-YOLO improves detection performance and robustness in complex fire scenarios while maintaining a lightweight architecture, indicating strong potential for deployment on edge devices.

## 1. Introduction

Fire, as a common and highly destructive disaster, poses a severe threat to warehouse environments characterized by dense personnel and concentrated materials. The development process of a fire can generally be divided into four stages: incipient, development, fully developed, and decay. Among these, the timely discovery and response during the incipient stage is the most critical link in fire prevention and control, which can effectively prevent the spread of the disaster and avoid massive casualties and huge property losses [1]. Therefore, constructing an efficient, accurate, and easily deployable early fire warning system for warehouses is of paramount importance.

Traditional early fire detection primarily relies on manual inspection and various sensors, such as temperature, smoke, and optical sensors. However, the accumulation of goods and the layout of three-dimensional shelves in warehouse environments make it difficult for conventional sensors to effectively monitor small-scale flame targets in hidden areas. Furthermore, achieving comprehensive monitoring in spacious warehouses requires the deployment of a large number of sensors, leading to high system costs and complex installation and maintenance. More critically, dynamic interference factors within the operational scene—such as frequent personnel movement, varying lighting conditions, surface reflections from goods, and equipment indicator lights—can easily trigger false alarms [2,3].

With the rapid advancement of computer vision and deep learning technologies, vision-based fire detection methods utilizing image recognition have gradually emerged. These methods identify flames by extracting features and employing classification models on real-time video streams captured by cameras. They possess advantages such as broad spatial coverage, rapid response, and flexible deployment, making them particularly suitable for fire warning tasks in complex, dynamic, or unattended environments [4]. Currently, vision-based fire detection methods are mainly categorized into hand-crafted feature extraction methods and deep learning-based methods [5]. The former identifies flames by extracting static features (e.g., color, texture, blurriness) and dynamic features (e.g., shape changes, flicker frequency, motion trajectory), combined with classifiers like Support Vector Machines (SVM) [6] and Random Forests [7]. However, such methods are extremely sensitive to illumination changes and background interference, resulting in limited adaptability and robustness. In contrast, deep learning methods have become the mainstream direction due to their ability to automatically learn and extract multi-level abstract features from large-scale data via neural networks.

Within deep learning approaches, the Transformer architecture has significantly advanced the vision domain. Vision Transformer (ViT) [8] partitions images into sequences and models them using self-attention mechanisms, achieving breakthrough performance in image classification tasks. Swin Transformer [9] further introduced a hierarchical sliding window strategy, realizing efficient fusion of local and global features and surpassing traditional CNN architectures on multiple object detection benchmarks. Nevertheless, Transformer architectures typically entail massive parameter counts and high computational overhead, making it difficult to meet the lightweight and real-time requirements of edge devices, thus limiting their application in fire detection.

In comparison, deep learning-based object detection algorithms can achieve a better balance between accuracy and speed by virtue of end-to-end training and automatic feature extraction capabilities. Detection frameworks are primarily divided into two-stage (e.g., Fast R-CNN [10], Faster R-CNN [11]) and single-stage (e.g., YOLO [12], SSD [13]) models. Although two-stage methods offer higher accuracy, they involve redundant computations such as candidate box generation, leading to slower inference speeds that are unfavorable for real-time applications. Conversely, the YOLO series of single-stage algorithms feature compact structures and rapid inference, demonstrating excellent performance and prominent application potential in fire detection tasks demanding high real-time responsiveness.

The practical requirement of limited computational resources on edge devices has driven researchers to focus on the lightweight design of network models. Existing studies have effectively compressed model sizes and computational costs by introducing depthwise separable convolutions, channel shuffling, and efficient feature fusion structures. For instance, Xu et al. [14] proposed Light-YOLOv5, which optimized feature fusion paths through a Light-BiFPN structure and effectively reduced the computational burden by combining improved depthwise separable convolutions and channel shuffling techniques. Fan et al. [15] constructed the F-ELAN module by integrating PConv with the Faster Block to enhance feature extraction efficiency. They also introduced GSConv and GSBlock in the neck network to strengthen feature representation while reducing computational redundancy, thereby improving edge device adaptability. Furthermore, YOLO-Fire, proposed by Xu et al. [16], utilized the SimpleC3 module to reduce model parameters and incorporated the Focal WIoU-loss function to enhance the recognition of irregular targets. Experimental results indicated improvements in both accuracy and real-time performance. However, in complex scenarios, flames often exhibit variable morphologies, blurred boundaries, and significant scale differences. Coupled with interferences from illumination changes, smoke occlusion, and specular reflections, visual fire detection effectiveness is often compromised, leading to missed detections or false alarms. Particularly in the incipient stage of a fire, flames typically manifest as small targets with weak feature representations, which are easily diluted or neglected during the downsampling and feature fusion processes. This severely impacts the timeliness and accuracy of early warning systems.

To address these challenges, researchers have attempted to improve model performance through structural optimization and feature enhancement. For example, Jiang et al. [17] proposed the DG-YOLO model based on YOLOv8, introducing a deformable attention mechanism to dynamically focus on flame shape features. It integrated a lightweight GSC2f module for cross-scale edge information fusion and designed a small object detector to capture texture details. Wang et al. [18] developed the FFD-YOLO model, which incorporated the LEIEM module into the C2f structure of the YOLOv8 backbone to enhance the perception of flame edges and spatial features. Additionally, it integrated SLSA and ECA attention mechanisms during the upsampling stage, improving multi-scale feature adaptability and achieving a 3.3% increase in detection accuracy over the original model. Furthermore, Huo et al. [19] proposed the MA-YOLO model based on YOLOv11, replacing the backbone network with a meta-feature extraction module and adjusting detection head scales to optimize adaptation to small flame targets. An adaptive feature fusion (AFF) mechanism was also introduced to suppress redundant information interference, resulting in a 9.8% precision improvement for small targets. Nevertheless, while these methods improve accuracy, they often increase network complexity, parameter redundancy, and computational overhead.

Although the aforementioned studies have made progress in fire detection, achieving high precision and robust detection in complex scenarios while satisfying the lightweight deployment requirements of edge devices remains a significant challenge. To this end, this paper proposes the FC-YOLO algorithm based on YOLOv11n. The main contributions are as follows:

1. The DySample dynamic upsampling module is introduced. Through a content-aware mechanism, it adaptively adjusts sampling point positions to effectively suppress noise amplification and improve the clarity of target boundaries. Furthermore, its point-sampling approach circumvents the heavy computational overhead typical of dynamic convolutions, achieving high-quality feature reconstruction without introducing redundant parameters.
2. The ADown lightweight downsampling module is adopted. By combining average pooling and max pooling with adjustable stride designs, it preserves global contours and local details for distant small targets. Simultaneously, by substituting traditional standard convolutions with this multi-branch pooling mechanism, it substantially reduces the model’s parameter count and computational complexity.
3. The Improved Efficient Multi-Scale Attention (IEMA) module is constructed. Integrating the inverted residual structure (iRMB) with the EMA attention mechanism, it strengthens the model’s focus on critical flame regions. By leveraging depthwise separable convolutions within the residual structure, this module achieves robust feature representation and cross-channel information interaction while strictly constraining the computational burden.
4. An additional small-object detection layer (P2) is introduced in the detection head. This mitigates the dilution of small target features caused by multi-layer downsampling, enhancing the recognition and perception of micro-scale flame targets, thus improving multi-scale detection precision and generalization performance.

## 2. YOLOv11 Algorithm and Optimization

**Figure 1:**
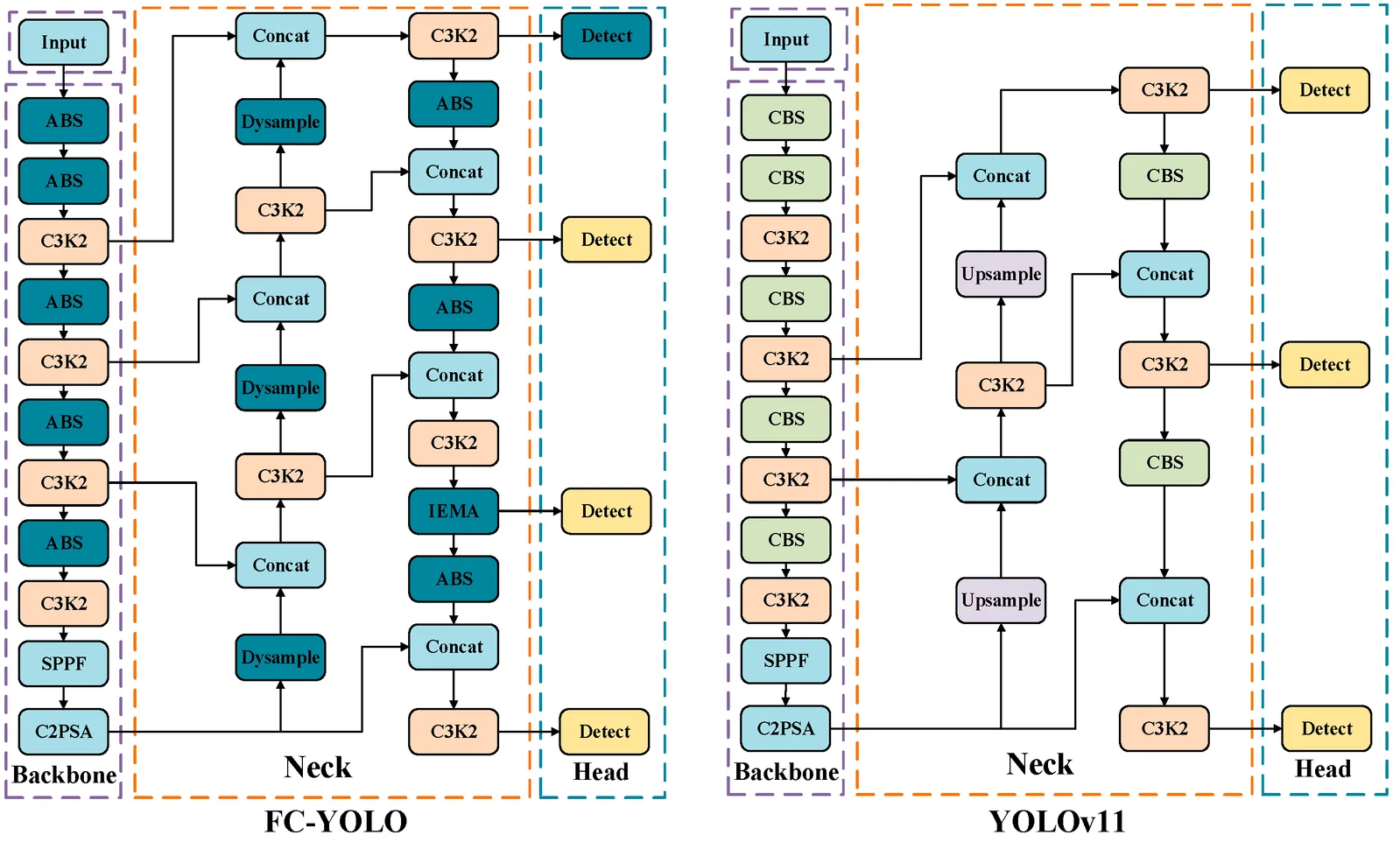
FC-YOLO and YOLOv11 network structures. The dashed frames indicate the division of the backbone, neck, and head network stages. Different colored blocks represent various neural network modules. Specifically, the dark blue blocks highlight the newly introduced modules in our proposed FC-YOLO, including the DySample, IEMA, and lightweight ABS modules.

The YOLOv11 algorithm series comprises five versions with progressively increasing parameter scales: YOLOv11n, YOLOv11s, YOLOv11m, YOLOv11l, and YOLOv11x. Among them, the lightweight version YOLOv11n demonstrates excellent performance while balancing inference speed and model complexity, making it the selected baseline model for this study. As illustrated in Figure 1, the YOLOv11n network structure primarily consists of a backbone network, a neck network, and a detection head. The backbone network is responsible for extracting fundamental image features, the neck network enhances the representation capability of multi-scale information through feature fusion, and the detection head executes target bounding box regression and category prediction based on the fused feature maps [20]. The structural optimizations of the proposed FC-YOLO network are mainly as follows: In the neck network, the traditional Upsample module is replaced by the DySample module; the CBS modules in both the backbone and neck networks are substituted with lightweight ABS modules (which integrate ADown, BatchNorm, and SiLU activation functions); the IEMA attention module is introduced into the neck network; and a small-object detection layer (P2) is added to the detection head.

### 2.1. Improved Upsampling Module

The neck upsampling module in YOLOv11n is utilized to restore high-resolution feature maps to enhance the model’s perception of target boundaries and shapes. In practical fire images, interfering factors such as smoke and uneven illumination frequently introduce random noise into the feature maps. During traditional upsampling processes, this noise is easily amplified, leading to blurred target boundaries and hindering precise localization of flame regions.

To overcome this, the DySample [21] lightweight dynamic upsampling module is introduced. DySample reconstructs the upsampling process using point sampling, generating optimized sampling point positions through offsets derived from input features. The dynamic sampling point generator accounts for the blurred edges and irregular shapes of flame regions. Considering that flames often exhibit high-frequency perturbations and discontinuous boundaries in images, dynamically adjusting the sampling grid allows the model to more accurately restore flame edges and contour details, effectively adapting to diverse and complex target morphologies in fire imagery. Furthermore, DySample’s point-sampling approach circumvents time-consuming dynamic convolution operations and additional sub-network generation, thereby reducing parameter counts and computational complexity. This effectively improves the quality of the upsampled feature maps, enabling enhanced model performance with minimal computational cost.

**Figure 2:**
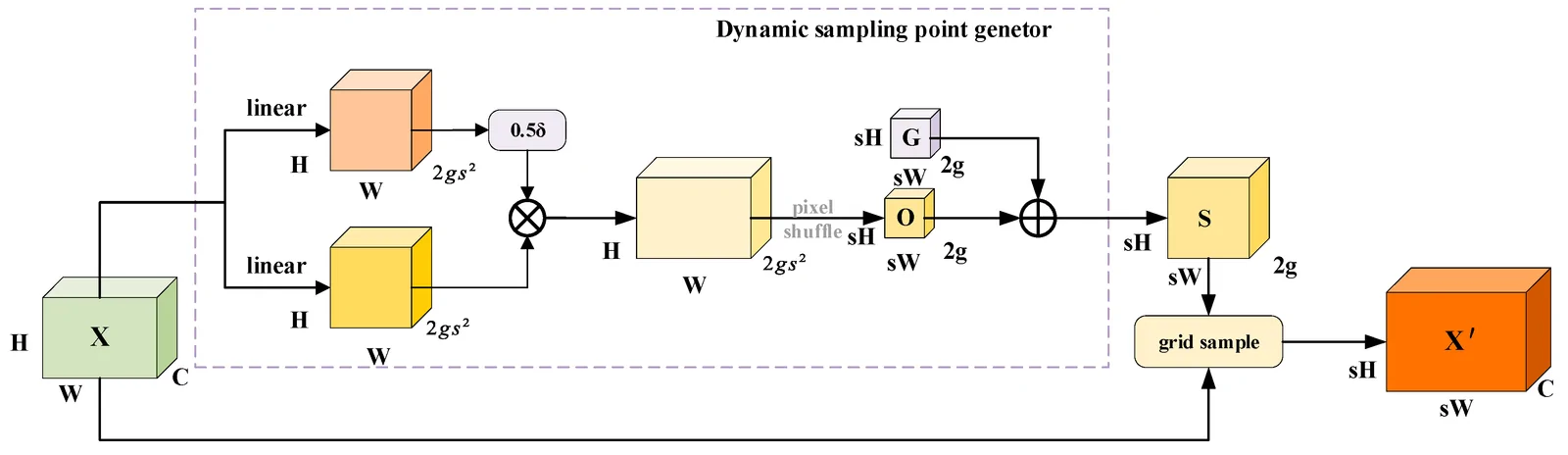
Dysample module structure.

As illustrated in Figure 2, the structure of the DySample upsampling module consists of a dynamic sampling point generator that processes the input feature map *X* to produce the upsampling offset *O*. This generator comprises a linear transformation and a Sigmoid activation function, with the calculation process formulated in Equation (1).

(1) $$O=0.5Sigmoid(linear_{1}(x))\cdot linear_{2}(x)$$

Subsequently, the offset *O* is added to the original sampling grid *G* to construct the sampling set *S*. Finally, the input features are resampled using the ‘grid_sample’ function to generate the upsampled feature map *X*′. Here, *s* denotes the upsampling factor, *S* represents the dynamic sampling set, *s*^2^ indicates the number of repetitions of the offset in each dimension, *g* signifies the number of groups, *G* is the original sampling grid, and δ represents the Sigmoid activation function.

### 2.2. Improved Downsampling Module

In complex fire scenarios, images frequently contain numerous distant small targets (such as incipient flames) which are prone to feature loss during downsampling due to weak feature representation. To bolster the model’s perception of small objects, the ADown [22] downsampling module is integrated into the backbone and neck networks of YOLOv11n, replacing traditional convolution operations.

**Figure 3:**
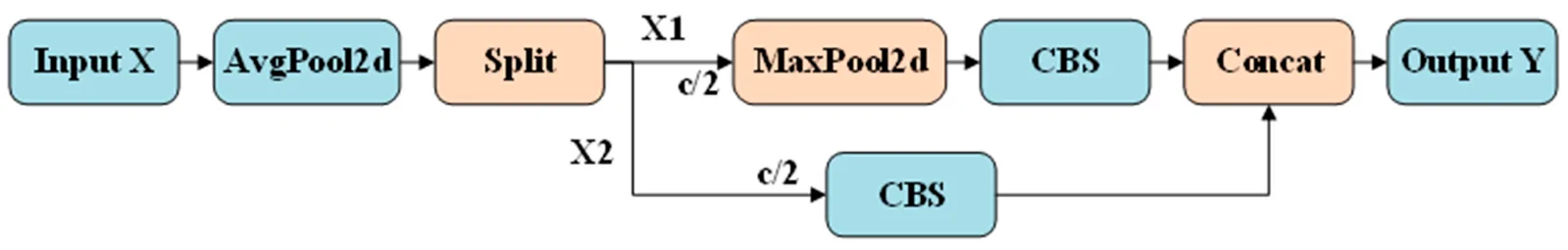
ADown module structure.

As illustrated in Figure 3, the ADown module first applies average pooling to the input feature map X to preserve the overall contour information of the flames. Subsequently, the feature map is split along the channel dimension into two sub-feature maps, denoted as X1 and X2. Specifically, X1 is initially subjected to max pooling to strengthen its response to localized abrupt regions (such as flame edges or high-brightness fire spots), followed by a 1 × 1 CBS module to compress the channel dimension and extract effective features. Meanwhile, X2 undergoes a 3 × 3 CBS operation. Finally, the outputs from both branches are concatenated along the channel dimension to generate the final feature map Y. Compared to traditional convolutional downsampling methods, this module preserves more effective information while reducing spatial dimensions through flexible stride control and a multi-branch structural design. Furthermore, it exhibits strong self-adaptive capabilities, enabling the dynamic optimization of feature extraction strategies across varying detection scenarios. Additionally, its lightweight design effectively reduces the parameter count, simultaneously lowering model complexity and enhancing detection precision.

### 2.3. Construction of the IEMA Attention Mechanism Module

Flame targets in fire images typically exhibit irregular morphologies, and the complex background interference renders feature extraction highly challenging. Attention mechanisms can effectively enhance a model’s capacity to capture critical regions and improve feature representation. To this end, a novel and efficient attention module, termed the IEMA module, is designed to strengthen the model’s perception and expressiveness regarding flames. This module draws inspiration from the inverted residual structure in iRMB [23] to extract features in a lightweight manner while maintaining low computational complexity. Simultaneously, by integrating the EMA [24] attention mechanism, it efficiently models information interactions across spatial and channel dimensions, thereby adaptively focusing on the critical features of flame regions.

**Figure 4:**
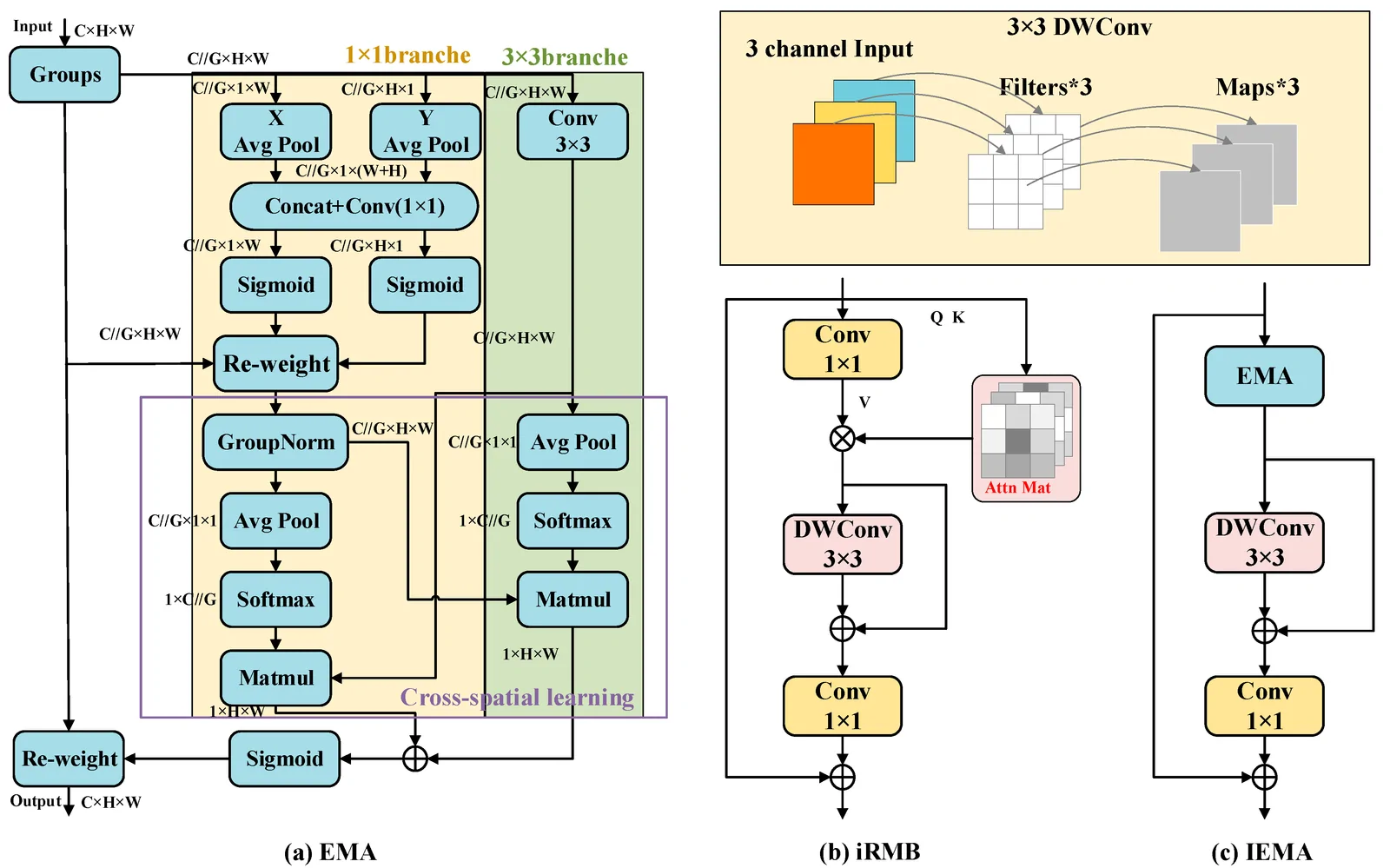
IEMA module structure. Asterisks (*) are used here to indicate the number of channels/dimensions.

As illustrated in Figure 4c, the overall structure of the IEMA module is based on a lightweight inverted residual design, integrating the EMA attention mechanism, a 3 × 3 depthwise separable convolution (DWConv), a 1 × 1 convolution, and multi-stage residual connections to strengthen the model’s perception of critical flame features. First, the input feature map is processed by the EMA module to focus on discriminative spatial regions, yielding a globally enhanced semantic feature map. Subsequently, this feature map passes through a 3 × 3 DWConv to further extract localized spatial information, which is then fused with the EMA output to achieve spatial multi-scale feature integration. The fused result is then fed into a 1 × 1 convolution to aggregate channel-wise information and compress redundant features. Finally, a residual connection links the output feature to the input to generate the final output. By virtue of this dual-level feature fusion and residual compensation mechanism, the proposed structure effectively enhances the representation capability and recognition robustness of flame targets in complex environments while maintaining its lightweight nature.

As illustrated in Figure 4b, the structure of the iRMB module first expands the channel dimension of the input feature map X via a dimension-expanding Multilayer Perceptron (MLP). Subsequently, a depthwise separable convolution (DWConv) is employed within this higher-dimensional space to extract complex spatial features and global dependencies. Finally, a 1 × 1 convolution is applied to reduce the dimensionality, followed by a residual connection with the original input to yield the final output.

As illustrated in Figure 4a, the structure of the EMA sub-module initially partitions the input feature map C × H × W into G sub-features along the channel dimension to learn diverse semantic information. Subsequently, three parallel sub-networks are employed to compute the attention weights for these grouped feature maps. Specifically, two sub-networks are situated within the 1 × 1 branch, while the remaining one is located in the 3 × 3 branch. In the 1 × 1 branch, global average pooling operations are utilized to capture precise positional information along the vertical and horizontal directions, respectively, as formulated below.

(2) $$Z_{c}^{H}(H)=\frac{1}{W}\sum_{0\leq i\leq W}x_{c}(H,i)$$

(3) $$Z_{c}^{H}(W)=\frac{1}{H}\sum_{0\leq j\leq H}x_{c}(j,W)$$

where x denotes the input feature, and c represents the channel index.

In the 3 × 3 branch, a single 3 × 3 convolution operation is performed to extract multi-scale features. Finally, two-dimensional (2D) global average pooling is executed on both the 1 × 1 and 3 × 3 branches, respectively. By fusing these multi-scale features and incorporating the Softmax function, cross-spatial information aggregation is achieved, thereby enhancing the feature representation capability within critical regions. The mathematical formulation of the 2D global average pooling is presented below.

(4) $$Z_{c}=\frac{1}{H\times W}\sum_{j}^{H}\sum_{i}^{W}x_{c}(i,j)$$

### 2.4. Small-Object Detection Layer

The original YOLOv11n architecture contains three detection layers, providing a certain degree of multi-scale detection capability. However, in fire detection tasks, flames vary drastically in morphology and span a wide range of scales. Small-scale targets are abundant. During multi-layer downsampling, the feature information of these small targets is easily lost, leading to increased missed detection rates and decreased accuracy. To alleviate this, a small-object detection layer (P2) is added to the detection head. By adjusting the anchor box dimensions and mitigating background interference strategies, this layer significantly enhances the detection precision and localization accuracy of small-scale flame targets in complex scenarios.

## 3. Experimental Results and Analysis

### 3.1. Dataset Construction

The performance of flame image recognition algorithms relies heavily on dataset quality. Currently, this field faces challenges such as insufficient sample sizes, imbalanced sample distributions, and monolithic backgrounds. Furthermore, publicly available video and image dataset resources remain limited, lacking authoritative standard datasets [24]. To address this, a flame image dataset was constructed using the following three approaches:

(1) Filtering high-resolution images from public datasets, including BoWFire [25], FASDD [26], and COCO2017 [27].
(2) Collecting and annotating high-quality fire images from the Internet via web crawler programs.
(3) Real-scene videos were acquired by conducting actual fire experiments and establishing simulated fire scenarios. Subsequently, image samples were extracted frame-by-frame from these video sequences to capture the morphological and transitional characteristics of flames in continuous scenes. This procedure provides robust data support for the model to learn the temporal dynamics and dynamic behaviors of flames.

#### 3.1.1. Sample Construction

Upon the completion of raw data acquisition, the dataset was subjected to manual screening and various data augmentation techniques to enhance sample diversity and improve the model’s robustness. These procedures specifically include the following.

**Figure 5:**
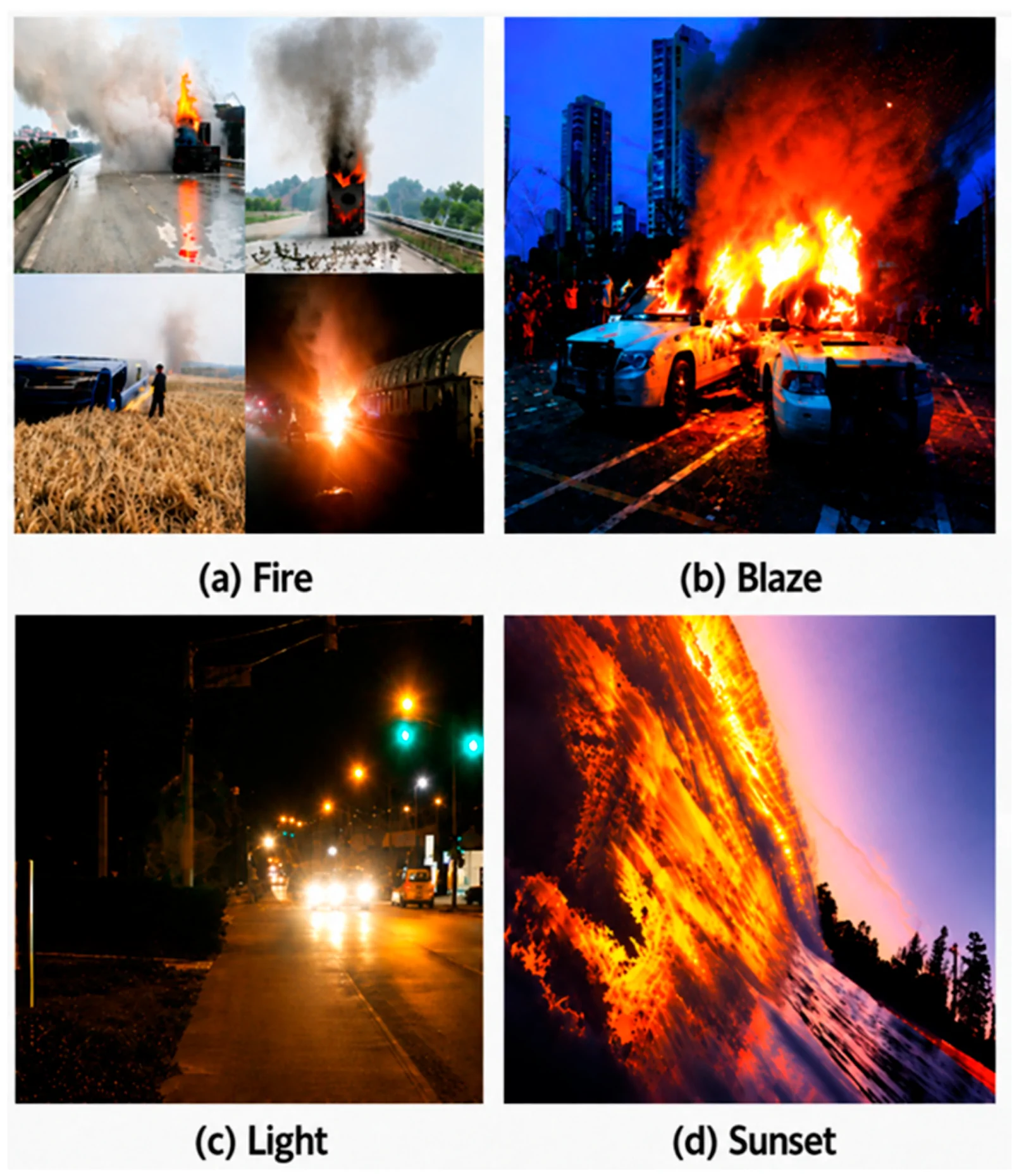
Examples of complex backgrounds and easily confusable objects introduced to mitigate false positive detections: (**a**) fire; (**b**) blaze; (**c**) light (negative sample); (**d**) sunset (negative sample).

(1) Inclusion of Complex Backgrounds and Negative Samples:

To enhance the model’s discriminative ability against easily confusable objects, non-flame images sharing similar color characteristics with actual flames—such as sunsets, red lighting, and reflective light sources—were additionally introduced into the dataset. Concurrently, a subset of pure background samples was supplemented. This strategy aims to enrich the diversity of negative samples and mitigate the risk of false positive detections, as illustrated in Figure 5.

**Figure 6:**
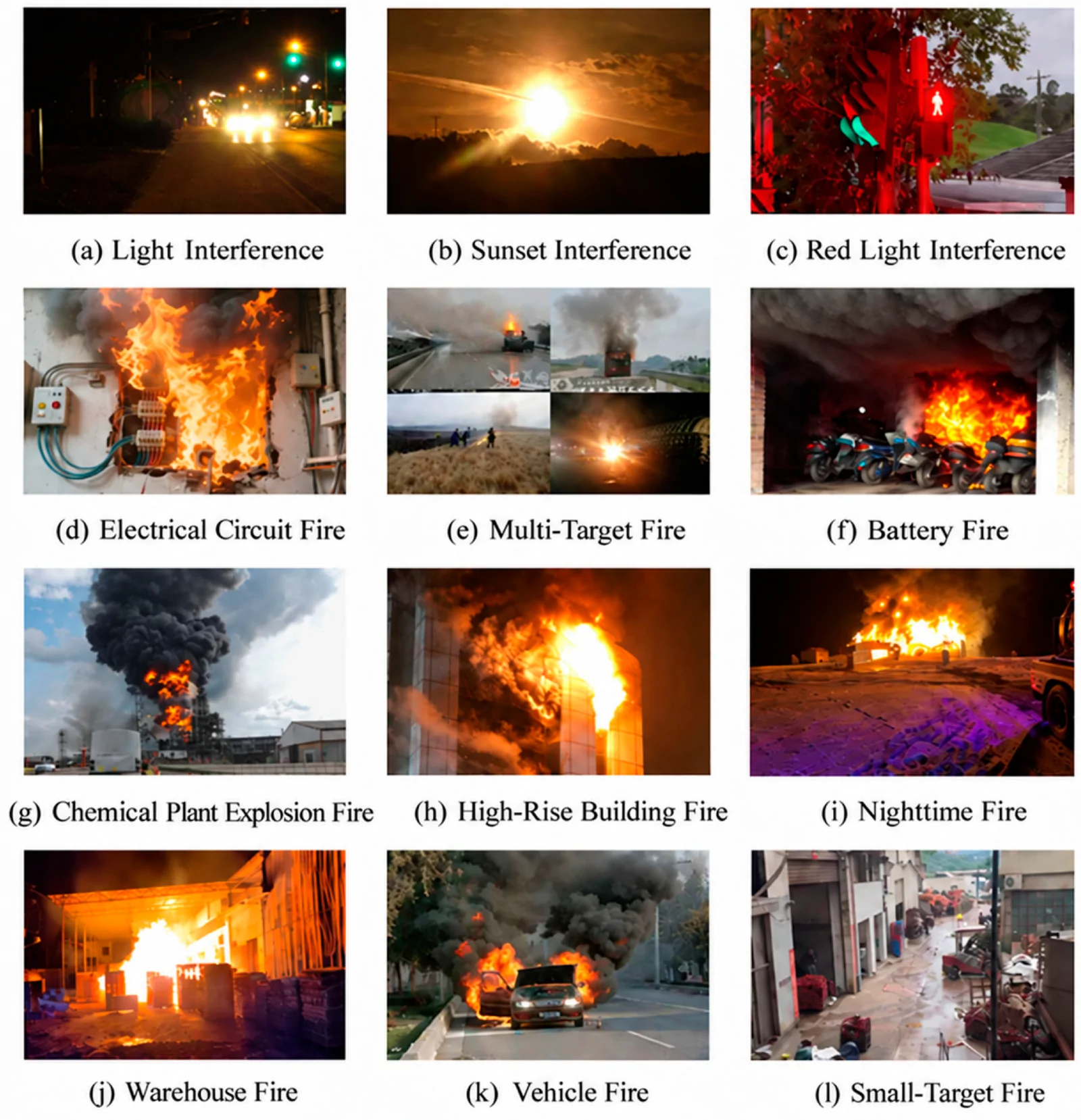
Selected image samples from the constructed dataset.

(2) Small and Multi-Target Sample Augmentation:

To address the challenge of small-scale flame targets during the incipient stages of a fire, a subset of images underwent scaling and concatenation procedures to generate composite samples containing multiple flame targets. This strategy effectively enhances the model’s perception capabilities regarding small and multi-target flames. To visually present the scene composition and sample diversity of the constructed dataset, Figure 6 illustrates examples of selected flame and non-flame samples.

#### 3.1.2. Data Annotation and Partitioning

During the data annotation phase, the LabelImg (version 1.8.6, Tzutalin, GitHub, San Francisco, CA, USA) tool was utilized to manually annotate the flame targets within the images, thereby ensuring the accuracy and consistency of the bounding box positions. Upon completion of the annotation process, the dataset was partitioned into training, validation, and testing sets at a ratio of 7:1.5:1.5. The detailed data distribution results are presented in Table 1.

To strictly prevent data leakage and ensure a rigorous evaluation protocol, images extracted from the same video sequence were grouped together and assigned exclusively to the same subset (i.e., entirely into either the training, validation, or testing set) rather than being randomly shuffled.

### 3.2. Experimental Environment and Evaluation Metrics

The model training phase was conducted on a personal computer (PC) equipped with an Intel Xeon CPU (Intel Corporation, Santa Clara, CA, USA) and an NVIDIA GeForce RTX 3060 GPU (NVIDIA Corporation, Santa Clara, CA, USA). The detailed experimental environment and the training parameters are presented in Table 2 and Table 3, respectively. To enrich the background context and improve the robustness of the model, Mosaic data augmentation was utilized during training. Furthermore, the model was initialized with pretrained yolov5s.pt weights using the PyTorch framework (version 2.0.0, Meta Platforms, Inc., Menlo Park, CA, USA). During the evaluation phase, the confidence threshold was set to 0.001 and the IoU threshold for Non-Maximum Suppression (NMS) was set to 0.60 to ensure a comprehensive assessment of the model’s Precision–Recall metrics.

To comprehensively evaluate model performance, Precision (P), Recall (R), and mean Average Precision (mAP@0.5 and mAP@0.5:0.95) were adopted as accuracy metrics. Additionally, model Parameters (Params), Floating-Point Operations (FLOPs), Model Size, and Frames Per Second (FPS) were introduced to assess computational complexity and practical deployment capabilities. The formulas are as follows:

(5) $$\mathrm{Precision}=\frac{TP}{TP+FP}$$

(6) $$\mathrm{Recall}=\frac{TP}{TP+FN}$$

(7) $$AP=\int_{0}^{1}P(r)dr$$

(8) $$FPS=\frac{Frames}{Time}$$

(9) $$mAP=\frac{1}{n}\sum_{i=1}^{n}AP_{i}$$

Here, TP (True Positive) denotes the number of samples predicted as positive that are actually positive; FP (False Positive) represents the number of samples predicted as positive but are actually negative; and FN (False Negative) indicates the number of samples predicted as negative but are actually positive. Params reflects the total number of model parameters, while FLOPs (Floating-Point Operations) measures the computational operations required for a single forward inference, serving to evaluate computational efficiency. In Equation (9), *n* represents the number of categories. Since this study exclusively focuses on the single category of ‘flame’, *n* is set to 1, which renders the values of mAP (mean Average Precision) and AP (Average Precision) identical. Furthermore, mAP@0.5 indicates the mean average precision at an IoU (Intersection over Union) threshold of 0.5. Meanwhile, mAP@0.5:0.95 covers the average mAP calculated across a range of IoU thresholds from 0.5 to 0.95 with a step size of 0.05. This metric imposes more stringent requirements on the accuracy of the predicted bounding box positions and scales, thereby more authentically reflecting the model’s robustness and detection precision in complex environments. Finally, FPS (Frames Per Second) signifies the number of image frames processed per second during the inference phase.

### 3.3. Ablation Study

To validate the contribution of each proposed module to the model’s performance, ablation experiments were conducted on the FC-YOLO model using the self-built dataset. Table 4 presents the performance metrics of the models under various module combinations, where A denotes the introduction of the DySample module, B represents the integration of the IEMA module, C indicates the adoption of the ADown module, and D signifies the addition of the small-object detection layer P2.

Based on the experimental results, following the introduction of the DySample module, mAP@0.5:0.95 increased by 2.3%, while mAP@0.5, precision, and recall also exhibited improvements. This indicates that the content-aware dynamic upsampling approach effectively enhances feature reconstruction quality and boundary localization precision, which is particularly crucial for detection tasks with higher IoU thresholds. Upon integrating the IEMA attention mechanism, precision, mAP@0.5, and mAP@0.5:0.95 rose to 92.9%, 88.4%, and 47.5%, respectively, demonstrating its capability to enhance feature focus on critical regions amidst complex backgrounds. Although the recall experienced a slight decline, this is likely because the attention mechanism, while emphasizing high-confidence regions, attenuated the model’s response to marginal or low-quality samples. After incorporating the ADown module, the model’s parameter count decreased significantly by approximately 19% (from 2.58M to 2.09M). Concurrently, the recall, mAP@0.5, and mAP@0.5:0.95 metrics all showed consistent improvements. This verifies that the ADown module serves as the core lightweight mechanism of the network. It effectively mitigates the computational burden and reduces model complexity while preserving the global structure and detailed information of the targets, thereby alleviating feature loss. Finally, following the addition of the small-object detection layer, the model achieved improvements across all detection metrics. Specifically, recall and mAP@0.5:0.95 increased by 2.7% and 3.4%, respectively, indicating that this layer significantly enhances the model’s perception of small-scale flame targets and mitigates the risk of missed detections.

When all four methods are combined, the model achieves its optimal comprehensive performance. Notably, the lightweight ADown module effectively offsets the minor parameter overhead introduced by the P2 small-object detection layer and the IEMA attention mechanism. As a result, compared to the baseline model, the final parameter count is still reduced by 14% (totaling only 2.20M), while precision, recall, mAP@0.5, and mAP@0.5:0.95 are improved by 1.8%, 4.6%, 2.4%, and 3.7%, respectively. This demonstrates an effective lightweight design that achieves a superior trade-off between detection precision and model complexity, making it highly suitable for edge deployment.

### 3.4. Visualization of Small-Object Detection Layer

**Figure 7:**
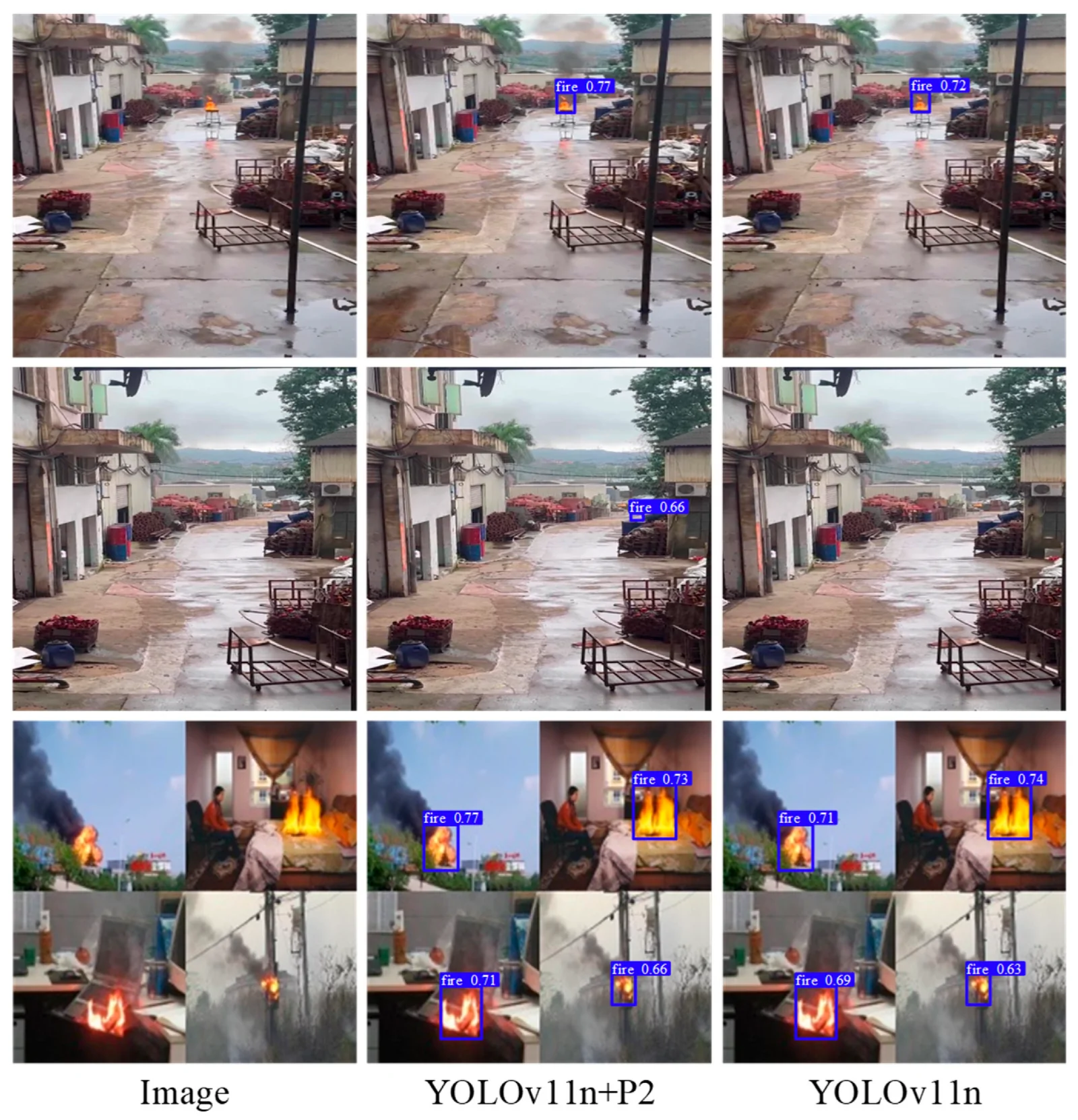
Visualization of small-object detection layer.

To validate the effectiveness of the small-object detection layer (P2), visual comparison experiments were conducted. As illustrated in Figure 7, following the introduction of the P2 layer, the model exhibits enhanced perception capabilities and detection robustness when confronted with distant, small-scale flame targets. This effectively mitigates the issue of missed detections for small objects and improves the recognition performance for multi-target flames within complex scenes. The incorporation of this detection layer significantly enhances the model’s practical utility in small-scale fire detection tasks, rendering it highly suitable for early fire monitoring.

### 3.5. Attention Mechanism Comparison Experiment

To validate the effectiveness of the IEMA attention mechanism in fire detection tasks, comparative experiments against various attention mechanisms were conducted on the self-built dataset. As presented in Table 5, the introduction of the IEMA module yields a precision of 92.9%, an mAP@0.5 of 88.4%, and an mAP@0.5:0.95 of 47.5%, while increasing the parameter count by less than 1%. Compared to other attention mechanisms, although iRMB obtains slightly higher mAP@0.5 and mAP@0.5:0.95 values, IEMA achieves the highest precision (92.9%) while maintaining highly competitive overall performance. This demonstrates an optimal trade-off, significantly enhancing the model’s feature modeling capability and recognition precision for critical regions within complex environments.

**Figure 8:**
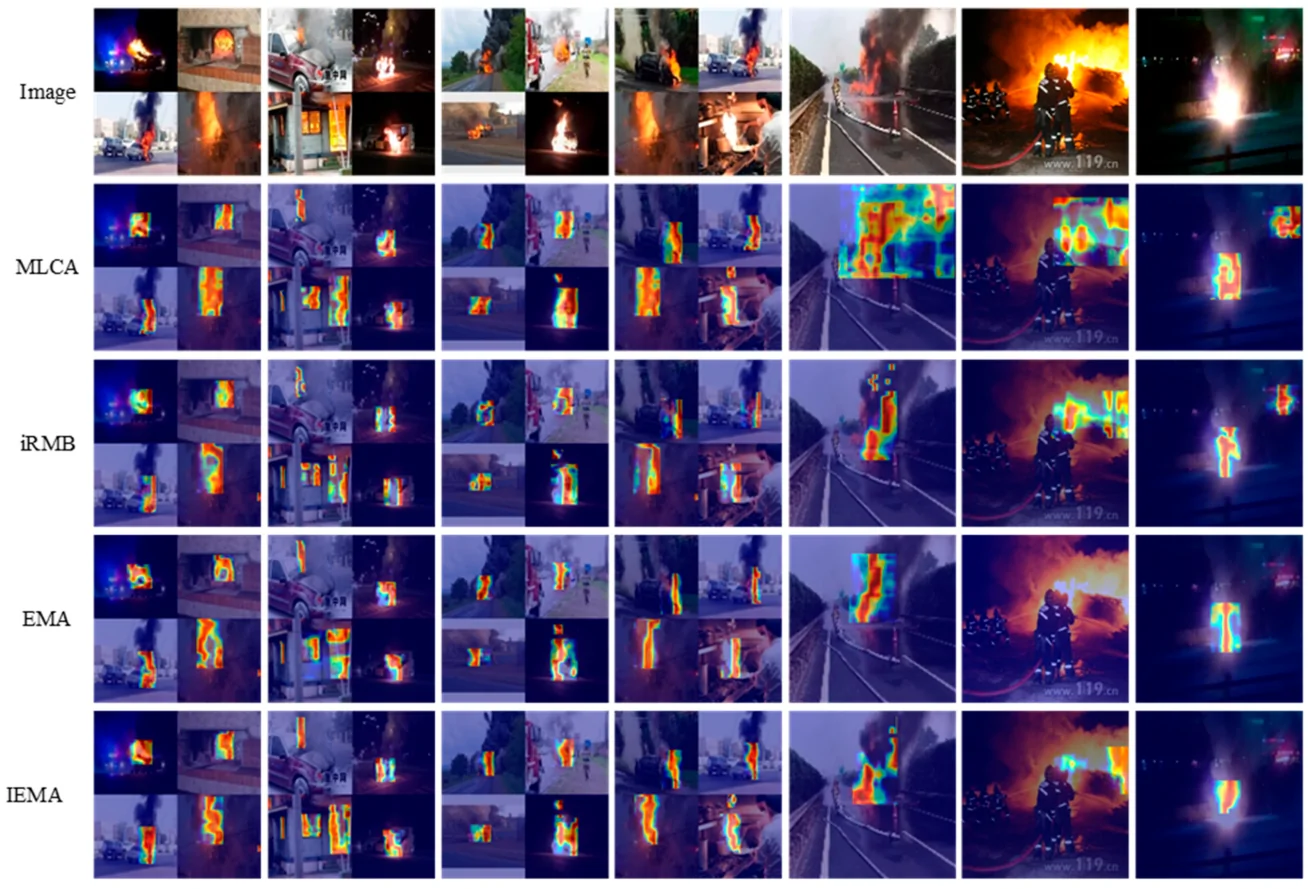
Attention mechanism comparison heatmap.

Furthermore, to further validate the response performance of the IEMA module on flame regions, heatmap visualization analysis is introduced to visually compare the feature response areas of different attention mechanisms. As illustrated in Figure 8, although MLCA and iRMB focus on flame regions, they still exhibit strong responses to certain irrelevant background areas, leading to attention dispersion. Moreover, they demonstrate inadequate modeling of fine-grained features such as flame edges and contours, which is prone to causing the omission of critical information and compromising detection accuracy. EMA possesses strong modeling capabilities in the spatial dimension with relatively concentrated regions of interest; however, it lacks sufficient perception when confronted with multi-scale flame targets, making it susceptible to information loss. In contrast, the IEMA module accurately focuses on the core regions and edge contours of flames across multiple samples, effectively suppressing the interference from irrelevant background information. It exhibits superior performance in spatial perception and feature representation, thereby further validating its effectiveness and robustness in complex fire scenarios.

### 3.6. Mainstream Algorithm Comparison Experiment

To comprehensively evaluate the overall performance of FC-YOLO, comparative experiments against current mainstream object detection algorithms were conducted on the self-built flame dataset, with the results presented in Table 6. In terms of computational complexity, FC-YOLO maintains a relatively low level at 9.1 GFLOPs, which is only marginally higher than those of lightweight models such as YOLOv5n and YOLOv11n-Fasternet. Nevertheless, it achieves optimal performance across key metrics, including precision (P), recall (R), mAP@0.5, and mAP@0.5:0.95, reaching 91.8%, 84.7%, 90.1%, and 50.0%, respectively. Furthermore, FC-YOLO features a minimal parameter count of just 2.20 M and a compact model size of merely 4.7 MB. This low overall resource overhead renders it highly suitable for deployment on edge devices.

Although FC-YOLO exhibits a marginally lower inference speed (FPS) compared to ultra-lightweight models such as YOLOv5n and YOLOv8n, its detection precision significantly outperforms them, demonstrating superior comprehensive detection capabilities and robust adaptability to complex environments. Furthermore, in comparison to high-precision models that incur substantial computational resource overheads—such as Faster-RCNN, YOLOv6, and YOLOv9s—FC-YOLO remarkably reduces both the parameter count and computational burden while maintaining high precision. These results underscore its prominent advantages in lightweight design and its substantial value for practical applications.

### 3.7. Baseline Visualization Comparison

**Figure 9:**
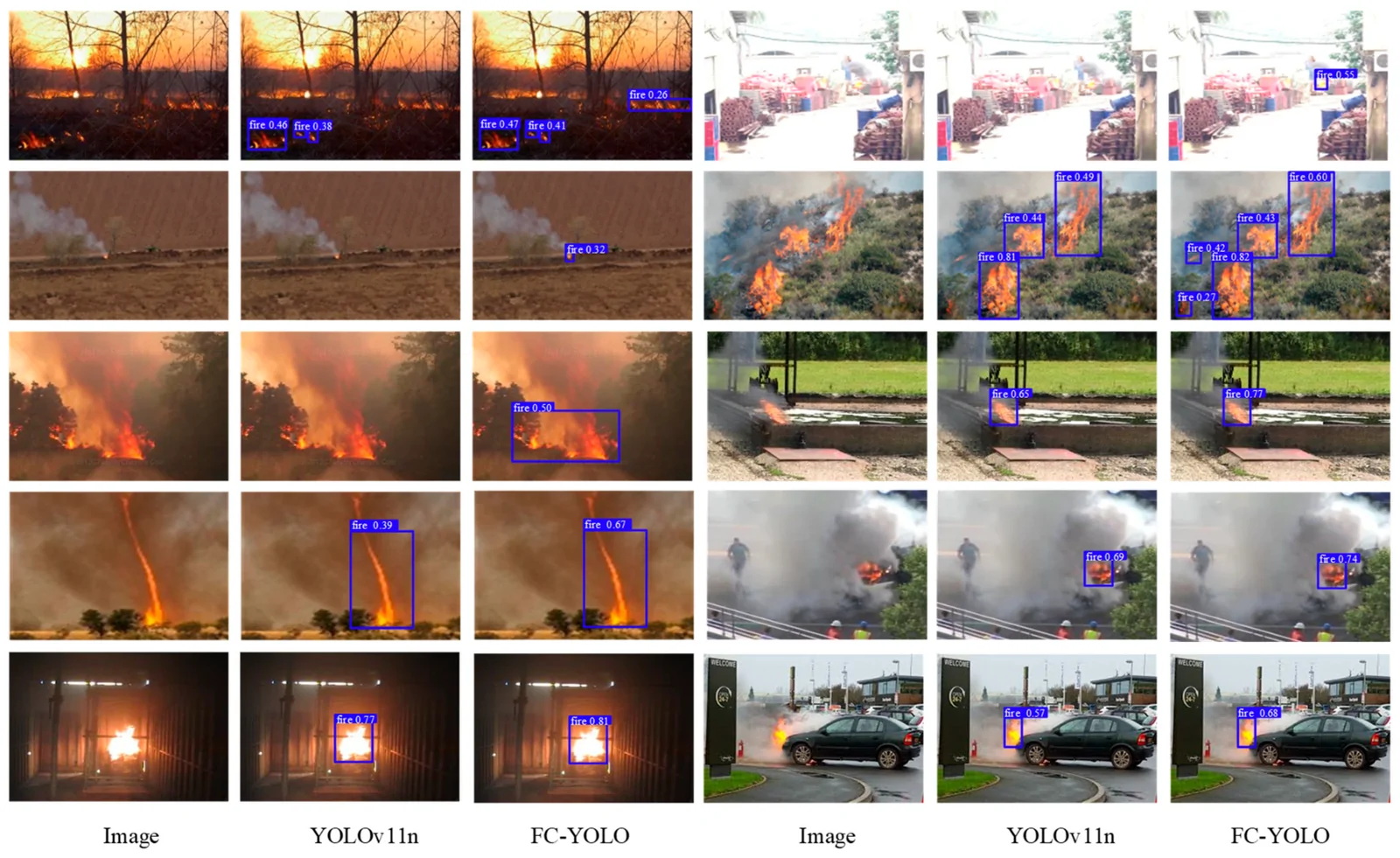
Baseline model visualization comparison.

To intuitively demonstrate the detection performance of the FC-YOLO algorithm, YOLOv11n was selected as the baseline for comparison, and visual experiments were conducted across a variety of typical complex environments, including sunset interference, strong illumination, small targets, dense smoke interference, complex indoor and outdoor lighting conditions, and target occlusions. As illustrated in Figure 9, YOLOv11n exhibits missed detections or relatively low confidence scores across multiple scenarios, demonstrating unstable detection performance, particularly under conditions involving multiple targets, small targets, or strong interference. In contrast, FC-YOLO consistently detects various flame targets and achieves higher confidence scores in the majority of scenarios, thereby thoroughly validating its robustness and practical utility in handling diverse and complex fire environments.

### 3.8. Generalization Experiment

To evaluate the generalization capability of the proposed model, two representative public fire datasets, namely the Home Fire Dataset [37] and the D-Fire Dataset [38], were selected for experimental validation. The Home Fire Dataset specifically targets indoor fire scene images, with an emphasis on capturing small-scale flames and early smoke features across diverse illumination and environmental conditions. It comprises a total of 6500 images, partitioned into 3900 for training, 1300 for validation, and 1300 for testing. The D-Fire Dataset serves as a comprehensive dataset for flame and smoke detection, encompassing varying smoke densities, diverse flame morphologies, and complex lighting conditions. It consists of 21,527 images in total, which are partitioned into 14,122 for the training set, 3099 for the validation set, and 4306 for the testing set.

As presented in Table 7, the experimental results of FC-YOLO demonstrate favorable detection performance across both the mAP@0.5 and mAP@0.5:0.95 metrics. Notably, it exhibits a significant improvement in the mAP@0.5:0.95 metric compared to other models. This indicates that the proposed model possesses strong generalization capability and robustness across diverse scenarios, enabling it to effectively adapt to complex and dynamic real-world fire environments.

## 4. Conclusions

In conclusion, this paper proposes FC-YOLO, an optimized early fire warning algorithm tailored for complex warehouse scenarios. By structurally refining the YOLOv11n baseline with dynamic upsampling (DySample), adaptive downsampling (ADown), an efficient multi-scale attention mechanism (IEMA), and a dedicated small-object detection layer (P2), the model effectively tackles the challenges of small-target recognition and background interference. Experimental results demonstrate that FC-YOLO achieves an mAP@0.5 of 90.1% and an mAP@0.5:0.95 of 50.0% on a custom dataset, while reducing the parameter count by 14% (totaling only 2.2M parameters) relative to the baseline. Ultimately, FC-YOLO delivers a highly robust, lightweight, and precise visual detection solution, exhibiting significant practical engineering value and strong potential for deployment in resource-constrained fire monitoring systems, including future warehouse safety applications. Future research will focus on further optimizing the network architecture design, exploring knowledge distillation, and applying quantization-aware training to further compress the model and accelerate inference speed on edge devices. Additionally, more adaptive data augmentation strategies and domain adaptation methods will be investigated to enhance the model’s generalization capability under severe environmental conditions, such as extreme illumination changes and dense smoke occlusion. These efforts aim to ultimately provide a more robust, efficient, and reliable visual detection solution for extreme scenarios.

## Figures and Tables

**Table 1:** Dataset distribution.

Dataset	Flame	Non-Flame	Total
Training	9527	4175	13,702
Validation	2016	1007	3023
Testing	2047	1126	3173
Total	13,590	6308	19,898

**Table 2:** Experimental environment.

Environment	Specification
Operating System	Ubuntu 22.04
CPU	Intel(R) Xeon(R) CPU E5-2650 v4
GPU	NVIDIA GeForce RTX 3060
GPU Memory	12 GB
CUDA Version	CUDA 11.8.0
Development Environment	Python 3.8
Deep Learning Framework	PyTorch 2.0.0

**Table 3:** Model training parameters.

Parameter	Value
Batch size	16
Image size (imgsz)	640
Epochs	200
Workers	8
Optimizer	Adam
Initial learning rate (lr0)	0.001

**Table 4:** Ablation experiment results.

Model	Params/M	P/%	R/%	mAP@0.5/%	mAP@0.5:0.95/%
YOLOv11n	2.58	90.0	80.1	87.7	46.3
+A	2.59	91.2	82.0	88.6	48.6
+B	2.60	92.9	79.1	88.4	47.5
+C	2.09	89.5	81.3	88.3	47.2
+D	2.65	91.7	82.8	89.5	49.7
+A+B	2.61	91.1	82.5	89.5	49.3
+B+C	2.11	88.0	79.8	87.3	47.0
+A+C	2.11	89.9	81.9	88.3	47.6
+A+B+C	2.13	91.1	82.3	89.4	48.2
+A+B+C+D	2.20	91.8	84.7	90.1	50.0

**Table 5:** Comparison results of different attention mechanisms.

Model	Params/M	P/%	mAP@0.5/%	mAP@0.5:0.95/%
YOLOv11n	2.58	90.0	87.7	46.3
+MLCA [28]	2.58	89.8	86.8	47.3
+iRMB	2.64	89.6	88.5	47.8
+EMA	2.58	91.0	87.7	46.6
+IEMA	2.60	92.9	88.4	47.5

**Table 6:** Comparison of mainstream algorithms.

Model	Params/M	GFLOPs	Model Size/MB	P/%	R/%	mAP@0.5/%	mAP@0.5:0.95/%	FPS
Faster-RCNN	136.6	369.7	108.2	38.1	76.5	67.8	-	13.5
SSD	23.6	174.8	90.6	91.7	69.7	86.1	-	42.5
YOLOv3-Tiny [29]	9.51	14.3	18.3	82.1	65.1	73.5	41.9	212.1
YOLOv5n [30]	1.87	4.6	3.6	83.2	77.3	85.0	46.0	105.6
YOLOv5s	7.02	15.9	13.7	87.2	78.7	86.5	47.8	98.8
YOLOv6 [31]	17.28	44.1	154	90.4	83.3	88.5	49.2	77.8
YOLOv7 [32]	37.19	105.1	71.3	69.2	68.8	74.7	37.6	78.3
YOLOv7-Tiny [33]	6.00	13.0	11.6	83.6	83.9	86.4	43.0	66.1
YOLOv8n [34]	2.68	6.8	5.3	87.3	81.1	86.4	45.2	91.6
YOLOv8s	11.1	28.6	21.5	88.2	80.7	87.7	49.1	84.0
YOLOv9s	9.92	40.6	77.5	89.1	82.9	89.0	49.8	44.2
YOLOv10n [35]	2.69	8.2	5.5	84.5	79.1	85.1	46.5	78.8
YOLOv10s	8.07	24.8	16.5	84.5	78.8	86.4	48.6	48.1
YOLOv11n	2.58	6.3	5.2	90.0	80.1	87.7	46.3	93.2
YOLOv11s	9.4	21.5	18.2	89.6	80.3	88.3	47.8	42.5
YOLOv11n-Fasternet [36]	2.37	5.5	4.8	89.7	81.3	88.5	48.1	83.2
FC-YOLO	2.20	9.1	4.7	91.8	84.7	90.1	50.0	73.4

**Table 7:** Comparison of generalization experiments.

Dataset	Module	mAP@0.5	mAP@0.5:0.95
Home fire dataset	YOLOv8n	89.0	55.2
	YOLOv10n	86.9	53.9
	YOLOv11n	89.0	55.4
	FC-YOLO	88.5	57.0
D-Fire dataset	YOLOv8n	78.4	43.8
	YOLOv10n	75.2	42.9
	YOLOv11n	77.4	43.7
	FC-YOLO	77.8	45.2

## Data Availability

The self-built flame dataset presented in this study is openly available in Zenodo at https://doi.org/10.5281/zenodo.22295784. The source code and pretrained weights of the FC-YOLO algorithm are not publicly available due to commercial confidentiality and pending intellectual property protections, but can be obtained from the corresponding author upon reasonable request.
